# Enhancing biomedical relation extraction through data-centric and preprocessing-robust ensemble learning approach

**DOI:** 10.1093/database/baae127

**Published:** 2025-05-22

**Authors:** Wilailack Meesawad, Jen-Chieh Han, Chun-Yu Hsueh, Yu Zhang, Hsi-Chuan Hung, Richard Tzong-Han Tsai

**Affiliations:** Department of Computer Science and Information Engineering, National Central University, No. 300, Zhongda Rd., Zhongli District, Taoyuan 320, Taiwan; Department of Computer Science and Information Engineering, National Central University, No. 300, Zhongda Rd., Zhongli District, Taoyuan 320, Taiwan; Department of Computer Science and Information Engineering, National Central University, No. 300, Zhongda Rd., Zhongli District, Taoyuan 320, Taiwan; Department of Computer Science and Information Engineering, National Central University, No. 300, Zhongda Rd., Zhongli District, Taoyuan 320, Taiwan; Department of Medical Research, Cathay General Hospital, No. 280, Sec. 4, Ren’ai Rd., Da’an Dist., Taipei 106, Taiwan; Department of Computer Science and Information Engineering, National Central University, No. 300, Zhongda Rd., Zhongli District, Taoyuan 320, Taiwan; Center for GIS, Research Center for Humanities and Social Sciences, Academia Sinica, 128 Academia Rd., Sec. 2, Nangang District, Taipei 115, Taiwan; Department of Medical Research, Cathay General Hospital, No. 280, Sec. 4, Ren’ai Rd., Da’an Dist., Taipei 106, Taiwan

## Abstract

The paper describes our biomedical relation extraction system, which is designed to participate in the BioCreative VIII challenge Track 1: BioRED Track, which emphasizes the relation extraction from biomedical literature. Our system employs an ensemble learning method, leveraging the PubTator API in conjunction with multiple pretrained bidirectional encoder representations from transformer (BERT) models. Various preprocessing inputs are incorporated, encompassing prompt questions, entity ID pairs, and co-occurrence contexts. To enhance model comprehension, special tokens and boundary tags are incorporated. Specifically, we utilize PubMedBERT alongside the Max Rule ensemble learning mechanism to amalgamate outputs from diverse classifiers. Our findings surpass the established benchmark score, thereby providing a robust benchmark for evaluating performance in this task. Moreover, our study introduces and demonstrates the effectiveness of a data-centric approach, emphasizing the significance of prioritizing high-quality data instances in enhancing model performance and robustness.

## Introduction

Biomedical literature is the foundational source of cutting-edge biomedical research findings. It encompasses a wide range of meticulously documented statistical and biological studies, distilled into simplified relationships between entities. With the exponential growth of biomedical literature, the utilization of relation extraction (RE) techniques becomes increasingly indispensable for managing and processing this abundance of data efficiently. RE methodologies adeptly identify entity pairs engaged in relationships and attribute specific relation types, thus enabling seamless unstructured text conversion into structured knowledge.

BioCreative [1]—Critical Assessment of Information Extraction Systems in Biology—is an organization that has been assessing the state-of-the-art in the fields of biomedical text mining and information extraction over the last few years. There is a challenge held by this group every year consisting of a variety of tasks. In this paper, we discuss Task 1, which is composed of two subtasks, in which we implement our approach in the BioRED [2] Track. In the first subtask, methods for extracting relations will be developed. The objective is to detect and categorize relationships that represent novel findings out of those that represent background, previously discovered knowledge or other available information. The second subtask requires the implementation of an end-to-end system that identifies the relationships in free text.

Through the use of ensemble learning and bidirectional encoder representations based on transformers (BERT), we investigate different preprocessing steps that will provide the highest performance for the system.

## Related works

In biomedical natural language processing, it is crucial to extract relations between entities. One approach involves defining relation candidates comprising entity pairs and their co-occurrence context, treating it as a text classification task. Traditional methods rely on rule-based or manually crafted features, while deep learning methods, offering automatic feature learning, show better accuracy due to their multilayer structure and ability to handle noise and uncertainty. However, deep learning’s performance suffers from limited supervised data, particularly in medical texts with sensitive information.

Pretrained language models (PLMs) like GPT [3] and BERT [4] excel in NLP tasks by leveraging large-scale unlabeled data during pretraining and fine-tuning with labeled data. Yet, general PLMs may falter in specialized domains like medicine. Domain-specific PLMs like PubMedBERT [5], pretrained on relevant corpora, outperform general ones in medical applications.

Fine-tuning PLMs for specific tasks face challenges due to discrepancies between pretraining objectives and fine-tuning setups, often leading to negative transfer and overfitting. Moreover, the need for labeled data limits performance in the presence of sample imbalance. Prompt tuning addresses these issues by aligning downstream tasks with pretraining objectives, improving efficiency, and interpretability. It transforms tasks into cloze tasks using templates with [MASK] tokens, with the PLM predicting the masked word, mapped to specific categories by a verbalizer [6] function. Typically, templates feature one [MASK] token, tailored to be understood in English. Ullah *et al*. [7] show that prompt-based fine-tuning improved the accuracy of text classification over standard fine-tuning approaches.

Several strategies have been explored to address labeled data imbalances in text classification. These include various data augmentation techniques as discussed by Azam *et al*. [8]. These approaches leverage pretrained language models, fine-tuning them on smaller datasets to boost their effectiveness in specific tasks. In the biomedical field, Lai *et al*. [9] have successfully employed a data-centric approach, demonstrating that merging datasets can enhance performance across five distinct relation extraction tasks, yielding promising outcomes.

Ensemble learning methods show promise in enhancing text classification performance. Ensemble learning is a meta-learning machine learning method that combines predictions from multiple models to achieve better predictive performance. In natural language processing (NLP), class imbalance problems are apparent in classification tasks such as sentiment classification, spam detection, and fake news detection. Ensemble learning methods have been shown to be effective in addressing these challenges in NLP tasks. Additionally, pretrained models such as BERT [4] and GPT [3] provide robust word embedding and have been used for text completion and dialogue and chat models in NLP tasks. Furthermore, incorporating ensemble learning with data augmentation methods can provide an effective way to augment training data for addressing class imbalance in NLP problems. Khan *et al*. [10] indicate the effectiveness of ensemble learning when combined with appropriate data augmentation techniques can significantly enhance performance on imbalanced datasets.

## Material and methods

### Data and tools

Dataset: The BioRED [2] corpus was provided, a collection of 600 PubMed articles that contains manual annotations of biomedical concepts and binary relationships by domain experts. For training, 500 articles are used, while for validation, 1000 articles are used. There are only 100 abstracts used for leaderboard evaluation.

PubTator Central API: A web service provided by the National Center for Biotechnology Information (NCBI). PubTator is a biomedical literature annotation tool that facilitates the extraction of key information from scientific articles. It automatically annotates documents with various biomedical entities, such as genes, diseases, chemicals, and more.

### Data-centric approach

We use a data-centric approach to improve the RE task by adding eight additional datasets collected from BioREx, Lai *et al*. [9], including BioRED [2], AIMed [11], DrugProt [12], DDI [13], HPRD50 [14], BC5CDR [15], EMU [16], PharmGKB [17], and DisGeNet [18].

### Preprocessing

By using the various preprocessing inputs, including prompt questions, entity ID pairs, and co-occurrence contexts, we improve the model performance. As shown in Table 1, special tokens and boundary tags can be added to enhance model understanding.

**Table 1.** The different preprocessing sentence pair inputs

Sentence pairs	Sentence 1		Sentence 2
	Relation type task	Novelty task	Relation type & Novelty tasks
**Baseline** (Tag, Entity)	-	-	Association between promoter −1607 polymorphism of <entity_type1> *entity1* </entity_type1> and <entity_type2> *entity2* </entity_type2> in Southern Chinese …
**Pairs1** (Tag, Entity)	<entity_type1> *entity1* </entity_tpye1> [MASK] <entity_type2> *entity2* </entity_type2> ?	<entity_type1> *entity1* </entity_tpye1> [MASK] <entity_type2> *entity2* </entity_type2> ?	Association between promoter −1607 polymorphism of <entity_type1> *entity1* </entity_type1> and <entity_type2> *entity2* </entity_type2> in Southern Chinese …
**Pairs2** (Tag, Entity)	What is [MASK] between <entity_type1> *entity1* </entity_type1> and <entity_type2> *entity2* </entity_type2> ?	What is [MASK] between <entity_type1> *entity1* </entity_type1> and <entity_type2> *entity2* </entity_type2> ?	Association between promoter −1607 polymorphism of <entity_type> *entity1* </entity_type1> and <entity_type2> *entity2* </entity_type2> in Southern Chinese …
**Pairs3** (Tag, Entity ID)	<entity_type1> *entity_id1* </entity_tpye1> and <entity_type2> *entity_id2* </entity_type2>	<entity_type1> *entity_id1* </entity_tpye1> and <entity_type2> *entity_id2* </entity_type2>	Association between promoter −1607 polymorphism of <entity_type1> *entity_id1* </entity_type1> and <entity_type2> *entity_id2* </entity_type2> in Southern Chinese …
**Pairs4** (Tag, Entity ID)	What is the relation type between <entity_type1> *entity_id1* </entity_tpye1> and <entity_type2> *entity_id2* </entity_type2> ?	What is the novelty type between <entity_type1> *entity_id1* </entity_tpye1> and <entity_type2> *entity_id2* </entity_type2> ?	Association between promoter −1607 polymorphism of <entity_type> *entity_id1* </entity_type1> and <entity_type2> *entity_id2* </entity_type2> in Southern Chinese …

Table 1 shows preprocessing pipelines that improve model performance. Each time, we provide two sentences: the first sentence (sentence1) is an entity ID pair or generated prompt question to provide semantic similarity for its corresponding pair; the second sentence (sentence2) corresponds to a co-occurrence context in which the entity is replaced by its corresponding entity ID and that entity is placed between two boundary tags (e.g. <GeneOrGeneProduct> and </GeneOrGeneProduct> for genes). These tags are kept as single tokens by adding them to the pretrained language model’s vocabulary. Moreover, we include the special tokens “[CLS]” and “[SEP]” at the beginning of the instance and in between sentence1 and sentence2, respectively, to conform to the standard practices of using pretrained BERT models for classification.

This study uses the state-of-the-art pretrained model PubMedBERT [5], which specializes in the medical domain, and fine-tunes it on the BioRED [2] dataset to form a text classification model. In the fine-tuning process, we pay attention to two aspects: the relation type and novelty of the relation. Each is treated as a separate task, so we create a classifier for each task: relation type and novelty. They are trained independently to provide confidence scores for each class. We can use these confidence scores to measure the model’s confidence in the predicted class for each instance.

### Deep learning model

**Figure 1.**
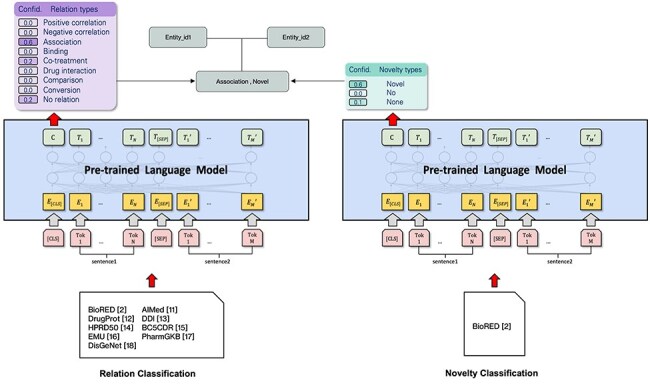
Model architecture.

The system used in subtask 1 is based on the BioRED [2] open source RE system implementation. Each instance of a relation candidate consists of two biomedical entities and their co-occurrence context. However, entity spans may be larger, and the relations with more than two entity IDs must be expanded to include those instances. The objective of this task is to classify the instances into predefined relation extraction types, or mark them as unrelated (i.e. “None”). This work also attempts to identify novel findings and existing information using the labels “Novel,” “No” (not novel findings), or “None” (negative examples). The model’s architecture can be found in Fig. 1.

**Figure 2.**
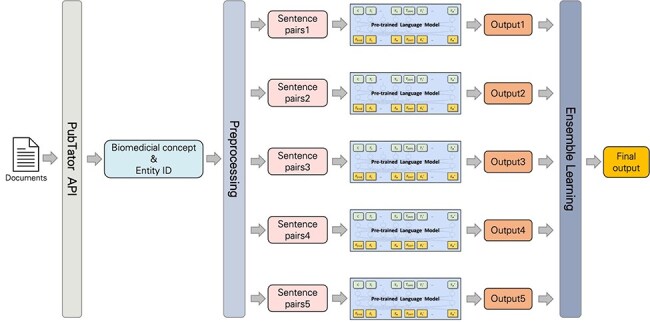
Workflow of our approach.

Subtask 2’s end-to-end system development leveraged PubTator’s API [19] to access and biomedical concepts and entity IDs during preprocessing. These concepts and IDs were then standardized to match dataset formats. To standardize the dataset provided by the PubTator API, certain terms were mapped to more general categories. For example, the expressions “ProteinMutation,” “DNAMutation,” and “SNP” should be transformed into “SequenceVariant.” Additionally, “Chemical” became “ChemicalEntity,” “Disease” turned into “DiseaseOrPhenotypicFeature,” “Gene” developed into “GeneOrGeneProduct,” and finally “Species” became “OrganisationTaxon.” Entity IDs were also transformed, including removing prefixes like “MESH:,” “tmVar,” and hyphens. We also generalized certain notations, like replacing “CVCL:” with “CVCL_” and ‘”S#” with “RS.” We made predictions on the processed output thanks to the pretrained models from subtask 1, and their performance was further improved by applying an ensemble learning method. The workflow is illustrated in Fig. 2.

### Ensemble model

The ensemble learning method’s Max Rule enhances system reliability and quality. This technique aggregates confidence scores from multiple models trained by using a different preprocessing input. The class with the highest probability score will therefore become the model’s final output as ensured by the Max Rule, which improves the robustness and accuracy of the classification system. However, there are situations where relations are found between entities, but the novelty was categorized as “None.” In this scenario, we replace the novelty type with “Novel,” which indicates the presence of new findings. Using these predictions, we generate the submission file.

## Ablation studies and discussion

Utilizing the BioCreative VIII BioRED Track CodaLab platform, the assessment score is computed. Subtask 1 encompasses three benchmark schemas: (i) entity pair: extracting the concept identifiers within the relation, (ii) entity pair + relation type: identifying the specific relation type for the extracted pairs, and (iii) entity pair + relation type + novelty: annotating the novelty for the extracted pairs. Subtask 2 introduces two additional benchmark schemas: (i) biological concepts (NER): recognizing the biomedical named entity, and (ii) ID: extracting the entity ID.

In our investigation, we systematically explored the influence of various model input formats on our text classification system performance. Initially, we introduced prompt sentences by integrating boundary tags and the token “[MASK]” between entity pairs (designated as pairs1). However, this modification did not yield statistically significant changes in performance metrics. Subsequently, we experimented with representing prompt text directly as pairs2, which resulted in notable performance enhancements of 2, 3, and 2% over the baseline across entity pair, relation type, and novelty benchmarks, respectively.

Further augmenting our exploration, we endeavored to normalize entity IDs by substituting entity names with unique identifiers within text instances (pairs3 and pairs4). While this adjustment did not elicit significant changes in relation type and novelty classification metrics, pairs4 exhibited a commendable 2% increase over the baseline, specifically in the entity pair benchmark.

Table 2 illustrates the significant impact of diverse information input strategies on our model’s performance across various metrics. In pursuit of model optimization, we meticulously curated sets of sentences demonstrating robust performance in both relationship identification and novelty detection. Subsequent application of ensemble learning techniques to these select sets led to a substantial improvement in our model’s efficacy. This approach emerged as a pivotal factor in achieving enhanced results, underscoring the importance of leveraging collaborative methodologies in text analysis endeavors.

**Table 2.** Performance on the test dataset of subtask 1

Inputs	Entity pair			+ Relation type			+ Novelty		
	P	R	F	P	R	F	P	R	F
Baseline	0.7107	0.7686	0.7385	0.5012	0.5419	0.5207	0.5325	0.5758	0.5533
Pairs1	0.7379	0.7412	0.7395	0.5223	0.5247	0.5235	0.5544	0.5569	0.5556
Pairs2	0.7327	0.7697	0.7508	0.5246	0.5511	0.5375	0.5502	0.5780	0.5637
Pairs3	0.7419	0.7259	0.7338	0.5445	0.5328	0.5386	0.5624	0.5502	0.5562
Pairs4	0.7423	0.7619	0.7520	0.5257	0.5396	0.5326	0.5513	0.5658	0.5585
+ Ensemble	0.7754	0.7488	0.7619	0.5769	0.5570	0.5668	0.6073	0.5864	0.5967
+ Data-centric	0.8002	0.7579	0.7785	0.6120	0.5796	**0.5953**	0.6219	0.5891	**0.6050**

P is precession, R is recall, and F is F1 score. Bold text represents the highest score for the main task.

Furthermore, our observations highlight the efficacy of a data-centric approach in improving overall model performance. By prioritizing the acquisition of high-quality data and employing collaborative techniques, we can effectively navigate the complexities inherent in text classification tasks, resulting in increased accuracy and efficacy.

## Top performing systems

Official results [20, 21] show that PubMedBERT alone achieves an F1 score of 0.7429 for Entity Pair identification, 0.5193 for Entity Pair + Relation Type classification, and 0.5625 for Entity Pair + Novelty Type classification. BioREx alone performs even better on Entity Pair identification (0.7568) and Entity Pair + Relation Type classification (0.5689). In contrast, GPT 3.5 and GPT 4 are significantly behind these models.

Top-performing teams in the competition employed diverse strategies. Team 129, for instance, developed a multitask system using PubMedBERT for context encoding and incorporating coreference resolution, entity pair identification, novelty identification, and relation extraction tasks. Their approach also leveraged adversarial training and model ensembles to boost performance and robustness, achieving F1 scores of 0.7559, 0.5667, and 0.5920, respectively, for the three tasks. This highlights the limitations of relying solely on a single preprocessing method, as our own system also benefited from an ensemble approach similar to Team 129.

Team 114 took a different approach, designing a unified model with BioLinkBERT to jointly classify relations and detect novelty. They employed techniques like surrounding entities with special tokens, utilizing a multihead attention layer, combining loss functions, negative sampling, and ensembling. Their best model achieved F1 scores of 0.7427, 0.5476, and 0.5854. While generally outperforming our single preprocessing method, their results fell short of our ensemble method. However, given their model’s strength, incorporating an ensemble technique could potentially further improve their performance.

Interestingly, Team 118 was the only team utilizing BioREx. However, their decision to augment training data with outputs from large language models like ChatGPT, Claude, and BingChat may have introduced noise-hindering BioREx’s performance (F1 scores of 0.7346, 0.5531, and 0.5645). Conversely, our new data-centric system with BioREx outperforms even our ensemble approach with PubMedBERT.

## Conclusion

Performances were significantly affected by different prompts and text representations. A change from naïve input of 73.85% for entity pairs to 75.20% for entity pairs with prompts and different text representations was observed. Novelty has been improved by 2%. Even when utilizing simplistic prompts and text representations across diverse configurations, sufficient diversity was maintained, allowing ensemble techniques to be effectively applied. This integration resulted in a notable improvement of ∼8% in novelty evaluation performance. Moreover, the model demonstrated even greater efficacy upon the implementation of a data-centric approach. Notably, performance metrics improved by an additional 1%, further emphasizing the efficacy of this methodology in enhancing overall model performance.

## Limitation and future work

To predict novelty, we rely on a relation-type prediction. If the relation type is “None” (no relation as a negative example), the novelty prediction will be ignored, resulting in a false negative outcome. In the case where the novelty prediction appears to be “None” (negative example), however, the prediction of relation type is not “None,” we will classify that novelty as “Novel” (novel finding), which will affect performance. As a part of our future research, we intend to apply some other techniques, such as the Hierarchical Bayesian approach, to solve the false negative issue.

## Data Availability

The BioRED track dataset is available at https://ftp.ncbi.nlm.nih.gov/pub/lu/BC8-BioRED-track/. The BioRED evaluation leaderboard is available at https://codalab.lisn.upsaclay.fr/competitions/16381.
